# InSpire to Promote Lung Assessment in Youth: Evolving the Self-Management Paradigms of Young People With Asthma

**DOI:** 10.2196/med20.2014

**Published:** 2013-05-21

**Authors:** Pierre Elias, Nithin O Rajan, Kara McArthur, Clifford C Dacso

**Affiliations:** ^1^Duke University School of MedicineDurham, NCUnited States; ^2^BSX AthleticsHouston, TXUnited States; ^3^The Abramson Center for the Future of HealthHouston, TXUnited States; ^4^Baylor College of MedicineHouston, TXUnited States

**Keywords:** pediatric asthma, chronic disease management, mobile phones, spirometry, gamification

## Abstract

**Background:**

Asthma is the most common chronic disease in childhood, disproportionately affecting urban, minority, and disadvantaged children. Individualized care plans supported by daily lung-function monitoring can reduce morbidity and mortality. However, despite 20 years of interventions to increase adherence, only 50% of US youth accurately follow their care plans, which leads to millions of preventable hospitalizations, emergency room visits, and sick days every year. We present a feasibility study of a novel, user-centered approach to increasing young people’s lung-function monitoring and asthma self-care. Promoting Lung Assessment in Youth (PLAY) helps young people become active managers of their asthma through the Web 2.0 principles of participation, cocreation, and information sharing. Specifically, PLAY combines an inexpensive, portable spirometer with the motivational power and convenience of mobile phones and virtual-community gaming.

**Objective:**

The objective of this study was to develop and pilot test InSpire, a fully functional interface between a handheld spirometer and an interactive game and individualized asthma-care instant-messaging system housed on a mobile phone.

**Methods:**

InSpire is an application for mobile smartphones that creates a compelling world in which youth collaborate with their physicians on managing their asthma. Drawing from design-theory on global timer mechanics and role playing, we incentivized completing spirometry maneuvers by making them an engaging part of a game young people would want to play. The data can be sent wirelessly to health specialists and return care recommendations to patients in real-time. By making it portable and similar to applications normally desired by the target demographic, InSpire is able to seamlessly incorporate asthma management into their lifestyle.

**Results:**

We describe the development process of building and testing the InSpire prototype. To our knowledge, the prototype is a first-of-its kind mobile one-stop shop for asthma management. Feasibility testing in children aged 7 to 14 with asthma assessed likability of the graphical user interface as well as young people’s interest in our incentivizing system. Nearly 100% of children surveyed said they would play games like those in PLAY if they involved breathing into a spirometer. Two-thirds said they would prefer PLAY over the spirometer alone, whereas 1/3 would prefer having both. No children said they would prefer the spirometer over PLAY.

**Conclusions:**

Previous efforts at home-monitoring of asthma in children have experienced rapid decline in adherence. An inexpensive monitoring technology combined with the computation, interactive communication, and display ability of a mobile phone is a promising approach to sustainable adherence to lung-function monitoring and care plans. An exciting game that redefines the way youth conduct health management by inviting them to collaborate in their health better can be an incentive and a catalyst for more far-reaching goals.

## Introduction

### The Global Epidemic of Pediatric Asthma

Asthma is the most common chronic disease in children worldwide [1], and its prevalence, morbidity, mortality, and economic burden have been rising in most countries for the past 4 decades [2-4]. Approximately 300 million people worldwide currently have asthma, and its prevalence increases by 50% every 10 years. In North America, asthma currently affects almost 9% of children (7 million in the United States alone) [5-9].

In developed countries, asthma disproportionately affects poor, black, and Hispanic children. Both the prevalence and the severity of the disease are greater in underserved populations. According to the Centers for Disease Control (CDC), US children in poor families (12%) are more likely than the non-poor (8%) to have asthma. Blacks are 3 times more likely to die from asthma than non-blacks. The impact of asthma varies through the stages of childhood; prevalence increases with age, but health care use is highest among the youngest children (ages 0-5) [9]. Frustratingly, recent emergency department (ED) use or hospitalizations are poor predictors of future use [10]. In under-resourced countries, morbidity and mortality from asthma tend to be disproportionately higher than in developed countries [4,8,11,12].

Asthma is a manageable disease that can be controlled through a combination of inhaled anti-inflammatory therapy and patient education. Effective self-management via individualized care plans has been shown to reduce the frequency of exacerbations and over-utilization of resources, and to increase quality of life [13]. However, despite 20 years of interventions, 50% of US children do not follow their care plans [13], leading to impaired quality of life, increased costs, and increased risk of asthma crises. The issue is not limited to the United States; most regions of the world have documented similar results [14-18].

### InSpire to Promote Lung Assessment in Youth (PLAY): Technology-Enabled Case Management

This paper presents the early development of a novel, user-centered approach to increasing children’s (aged 7 to 14) lung function monitoring and asthma self-care. The system was designed to help young people become active managers of their asthma through the Web 2.0 principles of participation, cocreation, and information sharing. Specifically, the InSpire system combines an inexpensive ($50-100), portable spirometer built with off-the-shelf components with a mobile phone and virtual-community gaming to help achieve widely accepted goals of asthma management, including objective lung function assessment and physician/patient partnership [3,19-21].

InSpire was designed to support these goals through an incentivized system that aims to encourage youth to monitor their lung function correctly while providing them with a toolset to help them follow their care plans. This toolset, housed on the compact mobile phone-spirometry device, comprises:

1. Games that incorporate spirometry maneuvers as core gameplay mechanics,
2. Quizzes for internalizing the child’s “Asthma Locus of Control,”
3. Geolocation to warn of certain asthma triggers in the child’s vicinity,
4. A chart that displays lung function over time and wirelessly sends readings to appropriate health professionals,
5. An account credit system that rewards youth for consistent lung function assessment over prolonged periods of time.

Ultimately, the goal of InSpire was to give children with asthma and their families an application for more consistent recording of lung function and comprehensive reference to their asthma care plans, as well as improved clinical data for physicians to use at point-of-care. This article describes the development process and initial feasibility testing of the prototype InSpire system.

## Methods

### Spirometry

**Figure 1:**
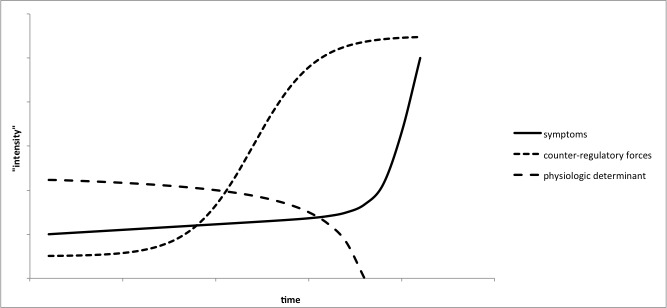
Relationship between symptoms, physiological determinant, and counter-regulatory forces. Symptoms of a decompensation event may not appear, although the physiologic alteration has been apparent for some time. Only when the counter-regulatory forces fail to compensate do symptoms appear.

Following a care plan requires the patient to have access to reliable, real-time data, yet children with moderate to severe asthma have a particularly hard time perceiving the reduction in lung capacity that signals that they are about to have an asthma attack [22]. That is not surprising, because, often, the alterations in physiology are subclinical and progress very slowly. For example, it is known that patients with asthma frequently have measurable declines in their lung function days prior to the clinical appearance of symptoms. The clinical symptoms are a result of a failure of the body’s compensatory mechanisms as shown in Figure 1.

In developing InSpire, we elected to use spirometry for lung function monitoring. Although the current standard for lung function measurement is to use a peak flow meter (PFM) and manually record readings in an asthma log, both the meters themselves and the log entries have been shown to have considerable reliability and validity issues, especially in pediatric patients. A 2011 study found that only 24% of school-aged children performed peak flow measurements correctly, and that was during a session where they knew they were being videotaped and assessed on the quality of their maneuvers [23]. Even when the meter was used correctly, the quality of PFM readings varied [24].

**Figure 2:**
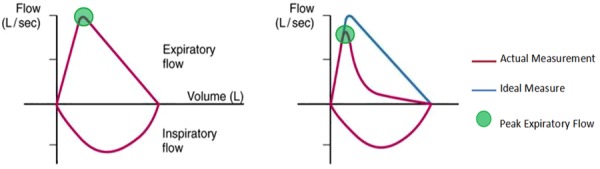
Representative spirograms of normal lungs (left) and those with emphysema (right). Peak flow meters report only the values centered in the green circles while spirometers provide more detailed data.

In contrast, spirometry has greater sensitivity to changes in small airways function, closer correlation to symptoms in children, and algorithms to determine quality of forced vital capacity data [24-28]. PFMs record only the peak expiratory volume while spirometers report the entire exhalation and inhalation process, as shown in Figure 2. In addition, spirometers electronically process incoming pressure readings and run quality-check algorithms to ensure data validity. However, until recently, spirometers were found only in clinical settings such as hospitals, were prohibitively expensive, and too cumbersome to be made portable.

### Spirometer Design

**Figure 3:**
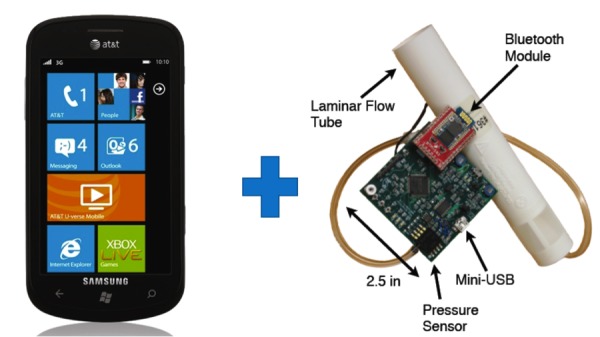
Rice mobileSpiro hardware and phone. The mobileSpiro processing block and laminar flow tube (which reduces the turbulence of the air flow). It runs off of 2 AA batteries and the spirometer connects with a mobile phone via Bluetooth. (Images courtesy of Microsoft Media Group and Rice University ECE).

We partnered with the Electrical and Computer Engineering Department at Rice University to develop a relatively inexpensive (US $50-100), portable, open-source spirometer that can be merged with a mobile phone (Figure 3). Mobile phones have enough computing power to process raw spirometry data and associated algorithms. In addition, they are a familiar form-factor for the target age group [29] and are rapidly pervading all levels of society.

The mobileSpiro architecture has 3 components: hardware (which samples, collects, and filters the raw data for transmission to the mobile phone), recording of the spirometry maneuver (where the raw data is converted into calibrated, meaningful flow measurements), and maneuver analysis (validation of the correctness of the maneuver). The hardware is Bluetooth-based and was developed in-house at Rice University’s Center for Multimedia Communication. The main processor is a Texas Instruments MSP430.

In order to keep costs down, all parts used in the spirometer are standard off-the-shelf components. In keeping with our design goals of portability and low power consumption, the spirometer runs on 2 AA batteries and, if it is used twice a day for up to 10 minutes a day, its batteries will last up to 80 days. The processor samples the data from the maneuver and transmits the raw samples to a Rayson BTM-182 Bluetooth module. The data received by the mobile phone is processed by low-level Linux drivers as part of the Android mobile phone data stack. Because different smartphones have different Bluetooth, mini-USB, and microphone input capacities, the Java software design enables collection of data via all 3 inputs [30].

The spirometer software housed on the mobile phone incorporates real-time validation algorithms. For each maneuver, the algorithms check for the following errors: cough, hesitation, glottis closure, and short-livedness. Each of these errors manifests itself in the flow-time and volume-time curves, and thus can be detected at the level of the mobile phone [30].

.

### Physician-Patient Partnership

Unfortunately, although spirometers hold the potential to improve asthma control and reduce asthma morbidity, most pediatric patients do not regularly monitor lung function on a daily basis [28,31]. Technological limitations are not the only hindrance to decreasing the number of sudden asthma attacks in children. Still, technology-enabled case management, in the context of a patient, parent, and physician partnership, has the capacity to provide better family preparedness and clinician access to patient data in the setting of acute decompensation [32]. The InSpire system was designed to help physicians and children with asthma develop such a partnership in 2 ways:

1. By promoting high-quality two-way communication between the provider and the patient/family by giving both parties accurate, actionable information.
2. By making the relationship a true partnership by increasing the child’s self-efficacy and motivation to monitor and manage the disease.

### Data-Driven Two-Way Communication

Current asthma treatment guidelines recommend that providers assess treatment adherence routinely and provide adherence promotion interventions as indicated [2]. InSpire aimed to automate this relationship through quality-checks of spirometry maneuvers, tracking of lung function over time, and two-way communication with the provider’s office via the mobile phone. In addition to the issues associated with preventive care, such as remembering to take medications consistently and correctly, treatment of pediatric asthma requires parents and children to make difficult decisions such as when to use different quick-relief medications to stave off or lessen acute attacks. The connectivity and quality-checked data of the InSpire system could possibly support such decision-making with physician approval.

### Helping Children Become Partners in Their Care

The provider-patient partnership is possible only when the child becomes a partner by recognizing the ability to take responsibility for managing his/her own health. A recent review of the research on children’s adherence to asthma management found that, while few interventions promoting adherence succeed, children who feel self-efficacy and hope about their asthma are more likely to follow their care plans [32].

**Figure 4:**
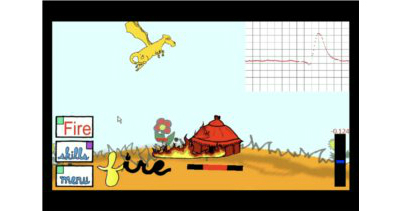
Image from the game housed on the mobile phone. The game integrates spirometry into the game play. Every correct spirometry maneuver causes Azmo the Dragon to breathe fire, earning the player points in the game. (Images courtesy of Microsoft Media Group and Rice University ECE).

We attempted to address these issues in the InSpire system by incorporating a game into the system. Drawing from design-theory on global timer mechanics and role-playing, the system aimed to incentivize completing spirometry maneuvers. For example, players defeat enemies in the game by making Azmo the Dragon breathe fire by correctly completing spirometry maneuvers (Figure 4).

**Figure 5:**
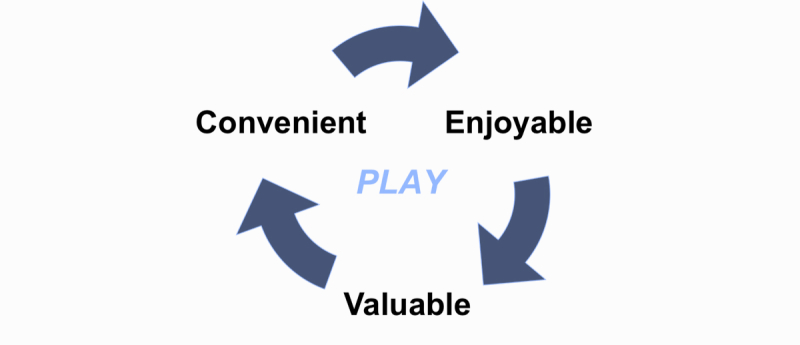
The goal of InSpire. By harnassing the power of mobile phones in the lives of children, InSpire aims to make asthma management more convenient (their phones are always with them), enjoyable (by turning assessment into a game), and valuable (by providing them with reliable, personalized, actionable data in real time).

The game was built in XNA on a Windows 7 phone and was designed to work on any Android phone. The phone can send the data wirelessly to clinicians and return care recommendations to patients in real-time. We based the game upon titles that are already popular with children aged 7 to 14 and that incorporate effective motivational frameworks. One example comes from Farmville, a simple game that has become enormously popular, with over 33 million unique active users. The game revolves around running your own farm and tending your crops, incorporating something known as a global-timer mechanic. The premise is that the user triggers an event, such as planting a certain crop. In a certain number of real-world hours, the crop must be harvested, or it will wilt and be lost. This has proven to be a powerful trigger [33,34] that motivated users to return to the gaming experience on a set schedule. InSpire incorporated similar scenarios where high-reward portions of the game involve spirometry maneuvers. Once these are completed, they cannot be revisited for 8 to 12 hours to prevent excessive use of the spirometer. By spacing these milestones in half-day intervals, we hoped to schedule children to complete their spirometry measures once at morning and at night. Thus InSpire was designed to address the 3 key elements of successful behavior change identified by Fogg (2007), motivation (through games and actionable data), ability (through the power and ubiquity of the mobile phone), and timing of triggers [33]. Our goal was to incorporate asthma management seamlessly into the child’s lifestyle (see Figure 5).

We hypothesized that such use of game design techniques and mechanics to engage children in their asthma monitoring would increase their adherence and self-efficacy of asthma care. Games are already used in health education to make mastering new skills and material more engaging. By taking advantage of children’s natural interest in gaming, this technique of gamification may encourage children to perform chores that they would ordinarily consider boring, such as monitoring lung function [34]. In 2004, the average US early adolescent spent about an hour every day playing electronic games [35,36]. Thus, early adolescents are already in extended daily contact with electronic games, which suggests that a carefully chosen game can attract and maintain youths’ attention, an essential prerequisite for affecting behavior change [33,37,38]. In fact, in children with cancer, gamification has been shown to increase adherence to regimens [39].

The InSpire system was grounded in social cognitive theory and the elaboration likelihood model, which provide models of learning for behavior change using video games [37,38]. Social cognitive theory indicates that behavior change is a function of enhanced skills and confidence (self-efficacy), while modeling and feedback are keystones for learning skills [40,41]. The elaboration likelihood model demonstrates that gaining and maintaining a person’s attention is the first step in promoting behavior change. Games add an element of fun, an aspect of both intrinsic and extrinsic motivation, which we anticipate will enhance behavior change through enhanced motivation [34].

The InSpire system was also designed to use the geolocation feature of most Android phones to warn children and their families of possible triggers (such as high pollen counts, ozone levels, and weather patterns) in real-time through an automatic text alert system tied to the United States National Weather Service. This data is freely available on their sites and updated daily, allowing for simple incorporation of certain asthma triggers into the application’s notification system.

Under-treatment of persistent asthma is common and contributes to impaired quality of life and increased risk for asthma crises. Conversely, over-treatment of asthma increases both medical costs and risk for adverse medication effects. InSpire tracks spirometry readings over time, stores them on a HIPAA-compliant server, and graphs them for interpretation. The ultimate goal was to help analysts develop better predictive models using data collected from daily spirometry measures to find earlier indications of acute decompensation and estimate long-term response on an individual basis.

### System Evaluation

The spirometer’s hardware and software algorithms were evaluated for their accuracy by comparing them with widely accepted clinical standards. The output accuracy of the spirometer hardware was tested versus an ISO13385 device, and the validation algorithms were tested alongside the CDC released NHANES III spirometry data set [30].

We tested the feasibility of the game via consultation with children from the target demographic of children aged 7 to 14. We chose this age range based on the age when children begin to acquire and use mobile phones [29], consultation with pediatric pulmonologists on the youngest age when children can function as active partners in their asthma care, and the age of interest for certain gaming mechanics. We tested the likability of the graphical user interface (GUI) as well as young people’s interest in our incentivizing system at a health fair sponsored by Texas Children’s Hospital. The fair was held in an under-resourced neighborhood of Houston with over 1500 children in attendance. Approximately 200 children played InSpire, and 9 children aged 7 to 14 with asthma more intensively tested the system and completed a survey on its interest and appeal after completing the informed consent process with a parent and the study staff. The survey and health fair observations were approved by The Methodist Hospital Research Institute Institutional Review Board. Future development will be guided by advisory groups of children from the target demographic recruited to take part in an iterative process of participatory design workshops.

## Results

The accuracy of the calibrated mobileSpiro sensor hardware was benchmarked against ISO13485-certified Thor PC FlowMeter, an established commercial spirometer. When the flow values from the mobileSpiro were compared to the Thor PC FlowMeter over a range of flows, deviation between the mobileSpiro and the FlowMeter never exceeded 8% across 0 L/s to 6 L/s [30].

The ability of the software algorithms to detect errors in spirometry maneuvers was tested using the NHANES III database [42] of raw spirometry curves (N=130,691). The curves were extracted from the database and run through the mobileSpiro validation algorithm. Each NHANES curve was scored on its validity and error type by a technician and by the NHANES computer software. The mobileSpiro successfully detected 95.5% (1141/1195) of cough errors, and 74% (4954/6695) of early termination errors. The false positive rate for the algorithms on these types of errors was 14-15% respectively. Inconsistencies in the NHANES III dataset prevented testing for hesitations [30].

Nearly 100% of the children with asthma surveyed (N=9) said they would play games like those in the InSpire system if they involved breathing into a spirometer. Two-thirds (6/9) said they would prefer the InSpire system over the spirometer alone, whereas 1/3 (3/9) would prefer having both. No children said they would prefer a conventional spirometer over the InSpire system.

## Discussion

### Addressing Adherence Through Meaningful Partnership

For two decades, the strategy for pediatric asthma prevention and control has consisted of 4 relatively simple goals: objective assessment, physician/patient partnership, control of environmental influences, and pharmacologic therapy through individualized care plans. Despite this clarity of purpose, asthma morbidity and mortality have only increased during the same time period, with one commonly cited reason being because patients and families find adhering to protocols difficult.

Using Medicine 2.0 principles of participation, cocreation, and information sharing, we have developed a mobile application that aimed to create a convenient central resource for asthma care. This central resource was designed to incorporate mobile spirometry with real-time feedback and the ability to send clinical information wirelessly. Additionally it was designed to host resources that notify patients about local asthma triggers using their geolocation. Lastly, it was designed to remind them of their asthma care-plan. We aim to provide a “one-stop shop” as part of daily use of mobile phones. Increasing children’s sense of partnership in their care and decreasing the barrier of access to quality, reliable, and personalized health information are necessary goals to achieving improved outcomes for the pediatric asthma population.

### Gamification of Asthma Management

Gamification is the use of game design techniques and mechanics to engage audiences and improve behavior-related outcomes. Nike+ and Nintendo’s WiiFit are two successful commercial examples where games have been used to promote fitness. The most successful example in research is FoldIt, a game that is designed so that players can manipulate virtual molecular structures that look like multicolored Tinkertoy sets. Since its inception, over 200,000 players registered and were able to solve a previously intractable protein-folding model in less than 10 days.

There is tremendous potential to apply gamification to other health problems, specifically chronic disease management, whereby the “chore” of adhering to prescribed regimens is replaced with captivating gaming. InSpire utilizes lung function measurement as a unique input device for mobile gameplay. The possibilities are vast, especially by utilizing ever-changing mini-games that produce a form of achievement. We hypothesize that this combination will keep the material fresh and leave children with a sense of accomplishment, further motivating them to perpetuate healthy behaviors.

### Mobile Phones as Persuasive Technology

Behavior-change researcher BJ Fogg stated in 2009 that “the mobile phone will soon become the most powerful channel for persuasion” [43]. Thus, we believe that today’s smartphones are a tremendous untapped resource for helping children to take responsibility for their health. In addition to having tremendous computing power, smartphones are highly attractive to children and mobile phones are a ubiquitous part of their lifestyles [44,45]. InSpire’s mobile-phone-based system fits Fogg’s definition of a persuasive technology with “captology” for children, combining as it does a tool for increasing abilities (spirometer, valuable data), a medium that provides experiences (gamification), and a social action function that creates relationships (connectivity and two-way communication with providers and, ultimately, other children with asthma) [38, 43,46] Thus, we hypothesize that enabling asthma management through InSpire’s persuasive technology will help to reduce barriers to adherence in 3 ways: convenience (access), fun (motivation), and value (see Figure 5).

### Predictive Analytics

The long-term temporal properties of airway obstruction in asthma have not been well studied. A 2005 article published in Nature noted,

It remains of interest to investigate if a combination of [airway measurements] could serve as predictors of a response to treatment. The ability to accurately and reliably predict individual long-term response would depend on the clinical end-points demonstrating both a small degree of variability within the study population and a large correlation coefficient between predictor and response variable. [47]

This problem has yet to be answered. Their specific call was for “multiple measurements over a length of time…to establish a more complete profile of response.” InSpire is a step toward developing a predictive model using data collected from daily spirometry measures to estimate long-term response on an individual basis.

### Limitations

The initial surveying of InSpire was limited by the small number of children with asthma (N=9) who participated. In addition, the long-term effects of the key feature “captology” or “stickiness” has yet to be tested. Future randomized controlled studies will address these issues as well as the system’s clinical effects.

### Conclusion

Previous efforts at home-monitoring of asthma in children have experienced rapid decline in adherence. An inexpensive monitoring technology combined with the computation, interactive communication, and display ability of a mobile phone is a novel approach to sustainable adherence to lung function monitoring and care plans. Gamification can redefine the way youth conduct health management by inviting them to collaborate in their health. This may better incentivize asthma management and ultimately may be a catalyst for more far-reaching goals.

### Further Research

We plan a pilot study of the prototype device to demonstration that the device is able to engage and motivate early adolescent children, generate valid data, and give them actionable information. Positive results from this trial would justify a larger randomized controlled trial to test its clinical benefit.

## Abbreviations

CDC: Centers for Disease Control
ED: emergency department
GUI: graphical user interface
PFM: peak flow meter
PLAY: Promote Lung Assessment in Youth
